# Sensitive Dentine and Its Remedy

**Published:** 1874-02

**Authors:** H. R. White


### SENSITIVE DENTINE AND ITS REMEDY.

BY H. R. WHITE.

Very much has been written and said upon this highly sensitive subject.

Many and varied have been the substances recommended from time to time, and as often discarded as ineffectual. With all that has been said and done, I may safely say it is the bane of every dentist, and the great stumbling-block to his success at the present day.

Is there then no substance that will effectually overcome this difficulty and that without injury to the teeth? As the results of my experience and practice for a series of years, and by comparing notes with others, who have used the same remedy and with equal success, I may emphatically say there is. That substance is ARSENIC, carefully and judiciously used, especially with a full knowledge of its effects.

Before saying more about this substance, which has been the cause of so much dispute, so many anathemas against any one who has dared publicly to acknowledge that he uses it, let us first look at what sensitive dentine is, and then consider how we may remove this condition.

We have in a tooth a pulp composed of blood-vessels and nerves, for support and vitality, like every other portion of the system. There is a deep, dense layer of dentine or bone covering this pulp, but penetrated by innumerable tubes and fibrilli through its substance, the fibrilli passing also into and through the interdental membrane and finally lost in the enamel, or outer dentine. Our tooth is now perfect in every respect. But from some cause, which we will not discuss at this time, decay takes place in the enamel; it gradually disintegrates and breaks away; at this time, or until decay has passed near to the junc-
o n of enamel and dentine, we have no sensitiveness. As the

*dentine* becomes exposed, or rather the *interdental membrane*, we find sensitiveness, and often in an extensive degree,—so much so that hot or cold air or drinks, or the touch of an instrument is exceedingly painful, thus often betraying decay when it would not be otherwise suspected or discovered, causing many to visit the dentist for examination. What, I ask, is the cause of this sensitiveness, and at this special point? As the decay progresses into the dentine and breaks down and destroys its substance, *this line*, if I may so speak, of sensitiveness disappears, and does not often occur until decay has nearly reached the pulp itself. Thus finding sensitiveness (only exceptionally) in the shallow cavities, far away from the pulp, or at the line of junction of enamel and dentine. My opinion is that disease has at this point exposed or so nearly exposed the sentient ends of the fibrilli or nerves which accompanied the tubuli, and are largely disseminated in the interdental membrane, the sensitiveness arising from exposure to air, fluids or instruments. Thus the term *sensitive dentine* is a *misnomer*. There is not, nor can be, any such thing. Destroy the pulp, and how much sensitive dentine or any other remains?

What is called sensitiveness, and which properly should be called pain, that is found in deep and large cavities, arises from near contact with the pulp itself, and is only found in proximity to the pulp, seldom in such cavities at the line of junction of enamel and dentine, unless we cut into and expose new layers of fibrilli. The pain thus found in deep cavities is very natural and expected, and of a different character from sensitiveness, as the nerves would be in a more highly sensitive state than in a tooth less decayed, especially as nature is endeavoring to prevent the farther inroads of disease by what is termed resistive action.

We come now to the question as to how is this sensitiveness to be disposed of without injuring the tooth. It is a well-known fact that many substances, such as nitrate of silver, cobalt, chromic acid, chloride of zinc, oxychloride of zinc and arsenic, will destroy and remove effectually this sensitiveness; yet alas, they will destroy the tooth also. The most effectual of all these substances is arsenic.

Prof. Tomes, in his last work, says "the most effectual of these is arsenic, and the fact that, after the deadened layer is removed, that below is found equally as sensitive as the first, is *proof conclusive* that it had not destroyed the pulp, but only the *layer* to which *applied*, and *no injury* thereby occurring to the tooth."

What a beautiful argument in favor of the much-abused arsenic, and from such a source; yet farther on in his work he again refers to it, viz : "Hence the use of arsenic is perfectly inadmissible when the cavity is deep, and can only be used for allaying tenderness of the layer of dentine immediately beneath the enamel." He should have said for destruction of fibrilli at the junction of enamel and dentine.

He says its use is to be deprecated although it is most effectual, and will only destroy the part applied to, and not injure the tooth. Now with a man of his opportunities, study and observation, I confess myself surprised at his conclusions, and fail to see even common sense in them. After telling how to use it, and that it produces just the result required, and the pulp not injured, he turns on himself and condemns without a reason, saving that it cannot be used in large, deep cavities without destroying pulp, is any dentist so thick or thin-brained as not to know this fact, and especially that in such large and deep cavities there is no necessity of applying arsenic, unless the nerve is intended to be destroyed ? again, on his own showing as to its needing only to be applied on the line of junction of enamel and dentine, there is no necessity of entirely discarding it.

Facts and repeated cases are stubborn things, and when accumulated for years, are the only reliable evidence upon which to base conclusions. For where one fails to-day, another by study, patience and perseverance attains the desired result to-morrow.

Had I never used arsenic, the mere reading of what Prof. Tomes says and its results as depicted by him, would certainly have induced me to give it a trial.

The truth is we have amongst the whole array of remedies, that have been and are used, but the one single substance ARSENIC, that can be relied upon as effectual, safe, certain and painless to the patient. I know whereof I speak—also well know the maledictions hurled by those who at least should know better, against any one courageous enough to use it. For at least ten years it has been my sole reliance, and have lost or discolored or injured but one tooth, and that only by foolishly yielding to the whims of my patient, and applying once too often, as Tomes remarks, in a deep cavity. Death of the nerve ensued ; it however taught me a good lesson—one I never forgot. I not only quote from my own extended practice, but from others, who have used it as directed, and as Tomes himself directs, and with the same unvarying result. Its application and time of action are simply a matter of brains and judgment. For every case proved to me of death of a tooth from its use, I will duplicate with any number from action of other remedies, showing that other substances are equally as dangerous as arsenic, and generally more so, as they are so carelessly used in general practice.

Here I may well be asked, how shall I use it ? 1st, with discretion and judgment. What do you wish to accomplish ? Simply to destroy the exposed ends of the fibrilli, by action of arsenic as a caustic. As soon as that is accomplished *remove it.* Why leave it to eat up and destroy all it can reach ? Remove it as soon as the object is accomplished. In burning a wart off a finger, would you continue until the finger was destroyed and so on up to the brain ? I think not. Effect the result, then remove the agent. *Use arsenic in the same way!*

It may be applied in any form most convenient. I always used the nerve-paste arsenic and morphine, equal parts, wet with carbolic acid or creosote, as most easily applied where desired, (viz. *on the line of junction of enamel and dentine,*) covered in with cotton or any substance to hold in place, there to remain from three to four hours, depending upon the density of thetooth. If a hard and dense tooth, four hours sufficed ; if a delicate, frail, translucent tooth, two hours. If the first application did not prove sufficient, apply again but for a less time. The patient, if allowed to go away from the office, must be made to distinctly understand when the drug must be removed and the dangers of neglect ; and if he cannot be trusted, should not be allowed to leave the office. Dealing with arsenic in this manner, and with as much care as you would use, not to expose a near nerve in excavating a tooth, no danger may be apprehended.

Should I find a deep cavity with these sensitive edges at its line of enamel and dentine, I place gutta percha in bottom first and arsenic only when required. A very minute quantity is all that is required.

After years of experimenting with a great variety of agents, my attention was drawn to arsenic by an article published in the Dental News Letter by J. D. White, giving his experience and results. I concluded that if he could use it, I could, and commenced experimenting. At that time I treated two of the worst cases that I ever saw or handled, and now more than ten years have passed away to my certain knowledge, the teeth remained as I left them. I have applied it to hundreds of teeth and never lost but the one referred to.

I should remark here that the first effect of the arsenic is to increase the sensitiveness; but by letting the tooth remain open for two or three days, this entirely disappears.

And now after ten years' constant practice, and that very extended, and in view of the results of others who are to-day using the *accursed arsenic*, I must say that carefully and judiciously used, as all things and agents should be in our profession, it is *certain*, *safe*, *reliable* and *painless*.

I am well advised of the rebukes which this article may call forth, from persons who call themselves FATHERS of DENTISTRY. But twenty-five years of constant practice, substantiated by facts, which are indisputable, make me fearless of their answers. If only one dentist is benefited by this article I am content.

